# Development and Validation of an Interdisciplinary Worker’s Health Approach Instrument (IWHAI)

**DOI:** 10.3390/ijerph16152803

**Published:** 2019-08-06

**Authors:** Lilian Monteiro Ferrari Viterbo, Maria Alzira Pimenta Dinis, André Santana Costa, Diogo Guedes Vidal

**Affiliations:** 1 UFP Energy, Environment and Health Research Unit (FP-ENAS), University Fernando Pessoa, 4249-004 Porto, Portugal; 2 Universidade Corporativa, Bahia 41745-002, Brazil; 3 CNPq Research Group “Dynamics of neuro-musculo-skeletal System”, Bahiana School of Medicine and Public Health, Bahia 40290-000, Brazil

**Keywords:** worker’s health (WH), interdisciplinary approach, questionnaire, instrument validation

## Abstract

The present study aimed to develop and validate an Interdisciplinary Worker’s Health Approach Instrument (IWHAI). The development stage comprised a group of 10 professionals, including physicians, nurses, nutritionists, dentists and physical educators, as well as a judges’ committee, composed by 19 recognized experts in the area of worker’s health (WH). For the validation of the IWHAI, the Spearman’s correlation coefficient (*r_s_*) was calculated, the factor analysis to the instrument was applied, and the Cronbach’s alpha (*α*) and the Intraclass correlation coefficient (*ICC*) were calculated. The IWHAI was structured in five dimensions, integrating 43 health indicators, on a scale of 0–4, totalling 215 sub-indices with closed response coding. The instrument was validated with a Kappa coefficient (KAPPA) (*k*), with excellent agreement for all attributes, i.e., *k* = 0.88 for applicability, *k* = 0.80 for clarity and *k* = 0.82 for relevance. *p >* 0.05 results reveal moderate to strong positive correlations between some variables, i.e., pests, vectors and air quality/drinking water quality *(r_s_* = 0.69). A total of 14 components of the factor analysis, explaining 62.6% of the data variance, were extracted. *α* value is considered moderate to high, *α* = 0.61, the *ICC* value also being considered moderate to high, with *ICC =* 0.61. The IWHAI is considered validated, constituting a technological innovation for an interdisciplinary approach in the field of WH, enabling the prevention and integral promotion of health.

## 1. Introduction

The 2030 Agenda for Sustainable Development, adopted by all United Nations Member States in 2015 [1] establishes 17 Sustainable Development Goals (SDGs) and defines integrated and indivisible goals balancing the three dimensions of sustainable development, i.e., economic, social and environmental. SDGs 3 and 8 relate to the health and labour aspects, aiming to ensure a healthy life and to promote the well-being for all, including the promotion of sustainable economic growth, inclusive and sustainable, full and productive employment and decent work for all, respectively [2,3]. The European Union (EU) Strategic Framework on Health and Safety at Work 2014–2020 [4,5,6] identifies important challenges and objectives including improvements in health and safety rules, prevention of occupational diseases and issues related to the aging of the workforce. Risk prevention and the promotion of safer and healthier conditions in the workplace are essential not only to improve the quality of employment and working conditions, but also to promote competitiveness [7]. Keeping workers healthy has a direct and quantifiable positive impact on productivity, contributing to the improvement and sustainability of social security systems [8,9].

As a disciplinary and professional field, the worker’s health (WH) covers the areas of medicine and engineering [10], which also incorporates epidemiology, administration, demography, statistics, ecology, toxicology, sociology, ergonomics and economics in the field of knowledge, comprising interdisciplinary theoretical practices [11,12]. In an extended perspective, the object of WH is the understanding of the health and disease process of human groups in their relation to work [13] and its potentiality is conditioned to the articulation between the two planes, i.e., health and disease [14]. WH involves distinct theoretical and operational fields around complex problems, thus being a privileged space for the formation of teams committed to interdisciplinary studies [15,16,17]. Environmental and sanitary situations are an example since they jointly involve the biological and physical environment, production, social organization, economy and culture in its interaction with human bodies and ecosystems, which may result in characteristics of greater health or vulnerability to risks [18]. Fragmented analysis of any of these variables, as performed by common science or even by multidisciplinary studies, would lead to major analytical and ethical problems involving limited intervention proposals [11,19]. WH surveillance is permeated by multiple, and sometimes conflicting, interests, in a permanent mechanism of transformation of the work process [20] and seeks to intervene in an interdisciplinary way in working conditions that negatively affect health, causing accidents or illness [11,16,21]. These actions must be linked to the daily life experienced by individuals, considering the environmental risks resulting from anthropogenic activities, as well as occupational hazards, mainly due to damaging conditions present in the work environments, affecting the health of workers, of their families and of populations living in the areas of influence of the productive units [22]. The associations between the environment as a whole and human health are very complex [23,24]. Intra-institutional articulation is the greatest obstacle to be overcome in the area of WH, as well as the need to improve the interface of the interdisciplinary team with proposals for interventions aiming to address ecological and occupational care [15].

A number of instruments have been developed to assess WH with the main objective of improving working conditions and promoting health and well-being in the workplace [25]. Examples are Health and Work Survey (INSAT) [26], Medical surveillance of exposures to occupational risks (SUMER) [27], Evolution and Workplace Health Relations (EVREST) [28] and the Basic questionnaire and methodological criteria for Surveys on Working Conditions, Employment, and Health in Latin America and the Caribbean (CTESLAC) [29]. In particular, the study by Yueng-Hsiang et al. [30] is a very important contribution to the development and validation of questionnaires applied in the WH and safety fields.

Prior studies [4,31,32,33,34,35] prioritize the analysis of working conditions in order to identify the risks related to work that impact on the health and well-being of individuals. It is of fundamental importance that other aspects of social determinants of health are included in these kinds of surveys, such as the social conditions in which people live and work [36]. Including these aspects would enable the development of health strategies to be more directed to the needs of the population. The potential of this systematized articulation based on intersectoral and interdisciplinary intervention as an action to transform work towards health promotion is broad, thus constituting an embryo of transformation in the WH theoretical–practical model.

The present study aimed to develop an Interdisciplinary Worker’s Health Approach Instrument (IWHAI) (Table S1) based on a theoretical framework involving the disciplines of medicine, nursing, nutrition, physical education and dentistry, as well as based on aspects related to social determinants of health [36,37,38], global disease burden [39,40,41], environmental aspects [23], SDGs [1] and, in particular, the working conditions affecting the health of the individual.

## 2. Materials and Methods

### 2.1. Study Design

This study is based on a strong methodological component, carried out from September 2017 to July 2018, in the WH service in the oil extraction and production industry in Bahia, Brazil. The study involved 10 health experts for the development of the IWHAI and its guidance manual. For the content validation, a judges’ committee comprising 19 recognized specialists in the area of WH, with at least five years of experience in an interdisciplinary approach, was also involved. A database comprised of a quota sample (*p* > 0.05), for 965 workers from a larger work population of 1275 subjects was chosen (Table 1). 

**Figure 1:**
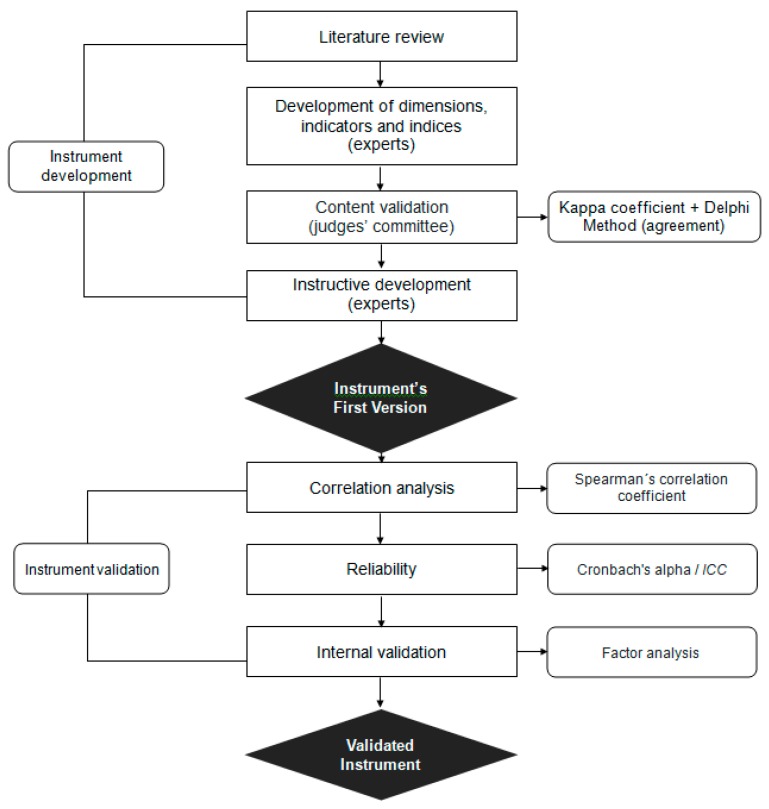
Interdisciplinary Worker’s Health Approach Instrument (IWHAI) development and validation process.

Figure 1 Detailed the Interdisciplinary Worker’s Health Approach Instrument (IWHAI) development and validation process.

### 2.2. IWHAI Development

The IWHAI development stage included a group of 10 WH experts, representing 20% of each profession, i.e., physicians, nurses, nutritionists, dentists and physical educators, all with more than five years of experience in the WH area. The literature review enabled the analysis of previously referenced instruments, as well as the theoretical framework for the development of a new instrument. IWHAI is methodologically based on the Quantitative Instrument for Sanitary Inspection (QISI) [42], both in terms of the process of developing and structuring the instrument, as well as in what relates the concept of potential risk. In its final version, IWHAI was structured with five dimensions, composed of 43 indicators, on a scale of 0–4, totalling 215 sub-indices with closed response coding. Each indicator was associated with an interval scale of 0–4, where zero represents non-existent or inadequate risk control and four represents optimal risk control, with the following graduation: 0—non-existent or inadequate; 1—tolerable; 2—reasonable; 3—good and 4—optimum. IWHAI proposes multidisciplinary assessments, encompassing an interdisciplinary approach. For each technical area, i.e., medicine, nursing, nutrition, dentistry and physical education, the main indicators of risk control for WH were defined. Finally, to reduce the subjectivity of the evaluator, the coding of closed answers for each sub-index of the scale was developed, with five possibilities for each indicator. Assessments comprised seven eight-hour meetings with professionals from each technical area and five meetings with the interdisciplinary team. For the classification of the indicators as critical and non-critical, a panel was developed with professionals, with each member giving an opinion about the indicator in question, thus obtaining a final group consensus. Of these indicators, 56.0% were classified as critical. At the basis of the IWHAI development, the need to establish a guidance manual that is able to assist health professionals in the task of filling each indicator was identified.

### 2.3. IWHAI Validation

To validate IWHAI content, a recognized 19 WH experts’ panel, i.e., judges’ committee, with minimum experience of five years in an interdisciplinary approach, was set up. A similar methodology, including statistical analysis, was used in other validation studies [43,44]. The first version of the IWHAI was presented to the group along with the spreadsheet for content evaluation, regarding the attributes of applicability, clarity and relevance, using a Likert scale. For each dimension, indicator and sub-index set, an eight-character code was created to organize the database generated in this step. Kappa coefficient (KAPPA) [43,45] was applied to analyse the results, considering *k* > 0.80–1.00, excellent agreement; *k* > 0.60–0.79, good agreement; *k* > 0.40–0.59, moderate agreement; *k* > 0.20–0.39, weak agreement and *k* > 0–0.19, no agreement. All results were accepted with KAPPA above 0.60, i.e., revealing good agreement. Following the Delphi method [46,47,48], already used in other health instrument development and validation studies, the 10 experts developing the draft instrument met again to review the judges’ recommendations. These were accepted and resulted in the exclusion of three indicators (“Chemical waste”, “Health waste” and “Bottled water”) and the inclusion of five indicators (Pests and vectors”, “Quality of air”, “Quality of drinking water”, “Work-related absenteeism” and “Work accident”).

### 2.4. Data Analysis

The procedures chosen to perform the validation of IWHAI were based on studies, namely those of Yueng-Hsiang et al. [30], Viterbo et al. [42] and Oliveira et al. [49]. After the selection of the dimensions and their respective indicators, the analysis of the relationship between variables was performed using IBM^®^ SPSS^®^ Statistics for Windows v.25.0 (IBM, Armonk, NY, USA) [50]. Spearman’s correlation coefficient (*r_s_*) was used in order to assess the correlation between ordinal variables. This test is indicated for non-parametric analyses, i.e., when there is no normal distribution or when the variables are not continuously quantitative, as is the case of the scale used in the sub-index. This test quantifies the relationships between the variables and their behaviour, either if linear or non-linear, positive or negative. For the validation of the construct, factor analysis was performed, i.e., analysis of the principal components of the correlations between variables. This technique assumes that the intercorrelations between the items can be explained by a smaller set of factors, representing relations between sets of interrelated variables. Through this analysis, the internal validity of the instrument was made, aiming to explain the variance of the results. This explanation was based on the independent components formed by a set of uncorrelated variables emerging from the transformation of correlated variables, obtained from the original variables. Several tests were used to assess the suitability of the respondent data for factor analysis. The Kaiser-Meyer-Olkin measure of sampling adequacy (KMO) test, measuring the data quality for the factor analysis, and the Bartlett’s test of sphericity, were used to verify if there is a relationship between the variables and if the matrix of correlations in the population is an identity matrix.

### 2.5. Ethical Approval

In all stages of the study, the recommendations and guidelines of Resolution 466/2012 [51] of the Brazilian Ministry of Health on ethical aspects regulating research with human beings, were followed. The study was approved by the Research Ethics Committee of the Bahiana School of Medicine and Public Health and CAAE no. 84318218.2.0000.5544. Before participating in the study, all subjects gave their informed consent for inclusion.

## 3. Results

In the validation stage of the IWHAI, men, aged between 51 and 60 years, with an administrative work regime, residing in the capital state (Salvador, Bahia, Brazil) and with a high school education, prevailed. The definition of the IWHAI dimensions and their respective indicators, as described in Section 2.2 is presented in Table 2.

The dimension, indicator and sub-index set form an IWHAI verification item. Table 3 shows an example of a “Nutrition” dimension verification item.

The IWHAI verification items were assessed for applicability, clarity and relevance. Using the KAPPA, the results show that the instrument has a high inter-observer agreement, as shown in Table 4.

The applicability attribute obtained 78% of the “I fully agree” option in the judges’ committee response. The clarity was evaluated as high, obtaining 87% of the answers. The relevance attribute corresponded to 88% of the answers in the “Important” option. The IWHAI was considered with validated content and excellent agreement for all attributes, presenting *k* = 0.88 for applicability, *k* = 0.80 for clarity and *k* = 0.82 for relevance, as shown in Table 4. The “Pests and vectors”, “Quality of air”, “Quality of drinking water”, “Work-related absenteeism” and “Work accident” indicators were not evaluated by the judges’ committee, since they were included by suggestion from the same experts, later accepted using the Delphi method.

Table 5 presents the *p* values, with only the correlations considered statistically significant at the 0.01 level to be shown. The analysis of Table 5 shows that the strongest positive relationships are between “Air quality” and “Pests and vectors” (*r_s_* = 0.69, *p* < 0.01), between “Physical activity level” and “Contemplation stage for physical activity practice” (*r_s_* = 0.78, *p* < 0.01), between “Oral hygiene quality” and “Periodontal condition” (*r_s_* = 0.79, *p* < 0.01), as well as between “Bodyweight” condition and “Energy balance intake” (*r_s_* = 0.59, *p* < 0.01). These are the variables in which the behaviour of both varies in the same direction, either increasing or decreasing.

The factor analysis presented in Table 6 reveals the adequacy of the sample through the means of the Kaiser–Meyer–Olkin measure of sampling adequacy (KMO) [52,53] (0.66 > 0.5) and through Bartlett’s test [54] (*x^2^* = 5252.03; *p* < 0.001). The factor loads are above 0.30, varying between 0.32 and 0.91, indicating a high level of validity of the selected items. Of the 43 indicators integrating the instrument, 14 components were extracted, which together account for about 62.6% of the total variance. All communalities have values above 0.40, showing the great proportion of variability of each variable that is explained by the factors. The measure of sample adequacy values suggests that the “Caries” indicator should be excluded from the factor analysis. Regarding the internal consistency of the IWHAI, it is observed in Table 6 that the global Cronbach’s alpha (*α*) is 0.61 and considered moderate/high [45,55,56]. With regard to the reproducibility of the instrument, the value of the intraclass correlation coefficient (*ICC*) is reasonable (0.61; 95% Confidence Interval = 0.562–0.652, *p* < 0.001).

## 4. Discussion

The current socio-economic context of the world demands from the companies the implementation of actions aimed at the improvement of living and working conditions, as well as the development of strategies for the promotion of WH, impacting in the reduction of absenteeism and medical expenses. For this, it is necessary to systematically monitor the population and implement programs aimed at reducing the potential risks of health, environment and work triad.

Similar studies resulting from the application of instruments, such as INSAT [25,26], SUMER [27], EVREST [28] and CTESLAC [29], focus on aspects related to working conditions. Other tools make it possible to calculate the epidemiological risk of each individual, such as the Framingham score [57] and the QRISK3 [58] calculator for estimating 10-year risk for myocardial infarction and stroke. The IWHAI works with the interdisciplinary approach and with the potential risk, which considers the possibility of occurrence of a health problem, without necessarily describing the health aggravation and the probability of occurrence. It is a concept expressing the value judgment about potential exposure to a possible risk [59], a clear advance in guaranteeing the prevention and integral promotion in the WH field.

The sample (Table 1) does not differ from the population at sex and age groups (*p* > 0.05), thus allowing for more robust analyses and conclusions. The results of the KAPPA (Table 4) show a consistent validity of the content, ranging in the applicability attribute between 0.79 and 1.00, in the clarity attribute between 0.71 and 0.93 and in the relevance attribute between 0.71 and 1.00. These values are much higher when compared to the reliability measurement of the Brazilian version of the Health and Work Survey INSAT-BR [60] (Brazilian adaptation) which showed KAPPA values ranging from 0.36 to 0.63, with a mean of 0.49. The results of the KAPPA in IWHAI are close to those of the QISI which, although developed for application in sanitary inspection in large food and nutrition services in Brazil, shows excellent agreement for the clarity (*k* = 0.82) and relevance (*k* = 0.92) attributes and good agreement for the applicability attribute (*k* = 0.78).

Regarding the correlations, there is an important association between the quality of the environment and the probability of occurrence of pests and vectors [23], as well as the close connection between oral hygiene and the oral health condition itself, as identified in past studies [61,62]. Additionally, the level of food knowledge is strongly associated with the level and quality of energy balance intake [7], with WH impacts and workers’ productivity impacts in general.

The 14 components extracted from the factor analysis (Table 6) are able to explain 62.6% of the phenomenon under study, i.e., the interdisciplinary approach in WH. This is a very satisfactory result since the dimension evaluated is abstract and influenced by several sub-dimensions. The results of the factorial analysis and the Cronbach’s alpha found in IWHAI are similar to the validation study by Yueng-Hsiang et al. [30], in which six components were extracted, able of explaining 47.9% of the data variance.

In this sense, the Food behaviour component (Table 6) stands out in the strong explanation of data variability (7.2%), revealing its importance in the interdisciplinary approach and in the workers’ own health [63]. The interrelation among the variables that integrate this component is visible because the level of food knowledge is directly related to the food intake, which in turn leads to changes in the individuals’ body condition and in their health condition, namely in altered blood pressure and altered glycemia. The environmental factors component emerges immediately afterward, with 6.0% explanation of the data variance. At this level it is important to emphasize the importance of a safe environment for the health of the worker that positively conditions the same, contributing to its promotion, rather than to its aggravation. Air quality is fundamental as it can lead to serious respiratory diseases, such as asthma, chronic pulmonary obstructive disease and lung cancer, as stated by Barreira et al. and the World Health Organization [64,65,66]. Water quality is also important because it is a direct transmission vector of diseases for the individual [67,68]. When these two indicators are bad, they can lead to the emergence of pests and vectors, which are highly negative for human and environmental health, as highlighted by Nazri et al. [69].

## 5. Conclusions

The development of the IWHAI enabled the collection of data by specialized teams using a single instrument that includes the health, environment and work triad. It also integrates the social determinants of health, as well as the risk factors studied as the main important ones for the global burden of disease. IWHAI content validation revealed excellent agreement for all attributes, with *k* = 0.88 for applicability, *k* = 0.80 for clarity and *k* = 0.82 for relevance. The reliability of the instrument is moderate/high (*α* = 0.61). Despite the indication to exclude the “Caries” indicator from the factor analysis, the authors decided to keep it in the instrument because of the importance it has in the WH assessment. The IWHAI development and validation demonstrates the possibility of applying an interdisciplinary approach in the WH field, with a focused performance of professionals of distinct specialties, as well as a mapping of intersectoral interventions, as an action to transform work towards health prevention and integral promotion. IWHAI application is thus found to be valid, robust and reliable.

### 5.1. Strengths and Limitations

The IWHAI is considered with validated content, being an innovation for the WH interdisciplinary approach in different labour contexts. Another important IWHAI expected contribution is the reduction of WH costs, considering that IWHAI acts simultaneously in disease prevention and health maintenance.

Although IWHAI is valid and reproducible, two main limitations must be considered, the need to maintain an interdisciplinary team able to respond to the various dimensions of the instrument and the existence of minimal environmental and health monitoring.

### 5.2. Future Applications

It is very important that new applications of the IWHAI be carried out so that its reproducibility is validated in other labour contexts. The validity of an instrument is also based on its availability and application by the scientific community. For this reason, the IWHAI is available as supplementary material and its application is free, provided that due credits are made to the IWHAI authors through the necessary citation of this article.

## Figures and Tables

**Table 1:** Population and sample characterization.

Sociodemographic Data	Population *n* (%)	Sample *n* (%)	*p*
**Sex**			
Male	1117 (87.6)	884 (91.6)	> 0.05
Female	158 (12.4)	81 (8.4)	
**Age Group**			
≤29	50 (3.9)	44 (4.6)	
30–39	350 (27.5)	261 (27.0)	
40–49	245 (19.2)	209 (21.7)	
50–59	556 (43.6)	410 (42.5)	
≥60	74 (5.8)	41 (4.2)	
**Total**	1275	965	

**Table 2:** IWHAI dimensions and indicators.

Dimensions	Indicators
Medicine	* Altered blood pressure * Altered glycemia * Arterial hypertension * Diabetes mellitus * Dyslipidemia * Musculoskeletal pathology * Psychiatric pathology * Stress level and symptoms * Tobacco use
Nursing	Air quality Drinking water quality * Ergonomic risks—physical aspects Ergonomic risks—organizational aspects * Exposure to environmental risks (physical, chemical and biological Family relationships Pests and vectors * Self-care level Social aspects—leisure Work accident * Work environment health conditions agents) Work-related absenteeism
Nutrition	* Alcohol use Altered triglycerides Bodyweight condition Energy balance intake Fibre intake Level of food knowledge * Saturated lipids intake * Simple carbohydrate intake * Sodium mineral intake
Dentistry	Bruxism * Caries Oral hygiene quality * Oral lesion on soft or hard tissues * Periodontal condition * Periodontal disease
Physical Education	Abdominal strength level Cardiorespiratory fitness * Contemplation stage for physical activity practice * Feeling of pain Flexibility level Manual gripping force Physical activity level

Note: * Critical indicators.

**Table 3:** Example of IWHAI nutrition verification item.

Dimension	Indicator	Indices	
Nutrition	Alcohol use	0	Frequent heavy drinker (drinks 1 time or more per week and consumes 5 or more doses per occasion, once a week or more)
		1	Frequent drinker (drinks once a week or more and may or may not consume 5 or more doses at least once a week, but more than once a year)
		2	Less frequent drinker (drinks 1 to 3 times a month and may or not drink 5 doses or more at least once a year)
		3	Non-frequent drinker (drinks less than once a month, but at least once a year and does not drink 5 or more doses at one time)
		4	Abstemious (drinks less than once a year or has never drunk in life)

**Table 4:** IWHAI inter-observer Kappa coefficient.

Dimensions	Rated Items	Applicability		Clarity		Relevance	
		*n_1_* (%)	*k*	*n_2_* (%)	*k*	*n_3_* (%)	*k*
Medicine	171	72	0.80	90	0.76	90	0.76
Nursing	285	73	0.86	81	0.78	79	0.78
Nutrition	171	71	0.79	89	0.71	90	0.71
Dentistry	114	90	1.00	84	0.93	90	1.00
Physical Education	133	86	0.95	90	0.83	90	0.83
*Mean*			0.88		0.80		0.82
(%)		78		87		88	

Note: Total of rated items = number of items of each dimension x number of judges’ committee; *n_1_* = percentage of items with the agreement in the high applicability criterion; *n_2_* = percentage of items with the agreement in the high clarity criterion; *n_3_* = percentage of items with the agreement in the important relevance criterion.

**Table 5:** Most significant Spearman’s correlation coefficients among variables under study.

Indicators	Pests and Vectors	Physical Activity Level	Contemplation Stage for Physical Activity Practice	Saturated Lipids Intake	Level of Food Knowledge	Body Weight Condition	Altered Triglycerides	Altered Blood Pressure	Abdominal Strength Level	Oral Hygiene Quality	Arterial Hypertension
Air quality	0.69 **										
Contemplation stage for physical activity practice		0.78 **									
Self-care level		0.40 **	0.34 **								
Sodium mineral intake				0.23 **							
Body weight condition					0.30 **						
Altered triglycerides					0.31 **						
Altered blood pressure					0.31 **	0.33 **					
Energy balance intake					0.59 **	0.59 **	0.37 **	0.37 *			
Flexibility level									0.39 **		
Periodontal condition										0.79 **	
Arterial hypertension								0.33 **			
Diabetes mellitus											0.35 **

* significant at 0.05 level; ** significant at 0.01 level.

**Table 6:** Factor load and communality of the indicators under study.

Component: %	Factor load														*C* *	(*α*) **
	I	II	III	IV	V	VI	VII	VIII	IX	X	XI	XII	XIII	XIV		
**Food Behaviour: 7.2**																
Energy balance intake	0.82														0.76	0.57
Level of food knowledge	0.58														0.58	0.59
Bodyweight condition	0.73														0.67	0.59
Altered triglycerides	0.47														0.52	0.58
Altered blood pressure	0.53														0.47	0.59
Altered glycemia	0.64														0.55	0.61
**Environmental Factors: 6.0**																
Pests and vectors		0.77													0.62	0.62
Air quality		0.91													0.85	0.61
Drinking water quality		0.55													0.57	0.62
**Oral Health:5.4**																
Oral hygiene quality			0.86												0.82	0.58
Periodontal condition			0.90												0.85	0.59
Periodontal disease			0.50												0.60	0.60
Bruxism			0.80												0.61	0.58
**Personal Factors: 5.0**																
Diabetes mellitus				0.68											0.59	0.60
Arterial hypertension				0.67											0.58	0.59
**Physical Activity: 4.9 **																
Physical activity level					0.85										0.77	0.59
Contemplation stage for physical activity practice					0.86										0.77	0.60
**Physical aptitude: 4.8**																
Cardiorespiratory fitness						0.48									0.57	0.60
Abdominal strength level						0.73									0.61	0.61
Flexibility level						0.67									0.59	0.61
Manual gripping force						0.59									0.62	0.61
**Musculoskeletal Factors: 4.6**																
Feeling of pain							0.82								0.71	0.61
Musculoskeletal pathology							0.82								0.71	0.61
**Behavioural Factors: 4.5**																
Simple carbohydrate intake								0.75							0.63	0.60
Fibre intake								0.67							0.61	0.61
Self-care level								0.48							0.62	0.59
**Mental Disorder and Working Conditions: 4.0**																
Psychiatric pathology									0.46						0.53	0.61
Work environment health conditions									0.55						0.46	0.62
Stress level and symptoms									0.66						0.64	0.62
**Consumption: 3.9**																
Alcohol use										0.67					0.59	0.60
Dyslipidemia										0.40					0.59	0.61
**Intake Levels: 3.8**																
Saturated lipids intake											0.57				0.62	0.61
Sodium mineral intake											0.59				0.57	0.61
**Organizational and Social Factors: 3.6**																
Ergonomic risks—organizational aspects												0.47			0.69	0.61
Social aspects—leisure												0.68			0.73	0.61
Work accident												0.53			0.60	0.61
Family relationships												0.80			0.68	0.61
Work-related absenteeism												0.82			0.72	0.61
**Occupational Risks: 2.5**																
Exposure to environmental risks (physical, chemical and biological agents)													0.61		0.48	0.61
Ergonomic risks—physical aspects													0.46		0.59	0.62
**Drugs and Injuries: 2.4**																
Tobacco use														0.77	0.66	0.61
Oral lesion on soft or hard tissues														0.32	0.58	0.61

Note: Extraction method: Principal components. Varimax rotation with Keiser normalization. Extraction criterion: Eigenvalues higher than one. Total variance explained by extracted components: 62.6%; KMO = 0.66; Bartlett’s test: *x^2^* = 5252.03, *p* < 0.001; * Communalities; ** Cronbach’s alpha (α) if item is removed; Global Cronbach’s alpha (α): *α* = 0.61; *ICC* = 0.61%—95% Confidence Interval = 0.562–0.652, *p* < 0.001.

## Supplementary Materials

The following are available online at https://www.mdpi.com/1660-4601/16/15/2803/s1, Table S1: Interdisciplinary Worker’s Health Approach Instrument—IWHAI.
