# Supplementary material for: Development and Validation of an Interdisciplinary Worker’s Health Approach Instrument (IWHAI)
Source: Int J Environ Res Public Health. 2019 Aug 6;16(15):2803. doi: 10.3390/ijerph16152803 (PMC6695998; doi:10.3390/ijerph16152803)
Supplement: Supplementary file 1 [file ijerph-16-02803-s001.pdf]

Interdisciplinary Worker's Health Approach Instrument - IWHAI

DIMENSION	INDICATOR	SCALE					CRITICITY
		Sub-Index 0	Sub-Index 1	Sub-Index 2	Sub-Index 3	Sub-Index 4	
**Physical Education**	Physical activity level	Sedentary	Irregularly Active B	Irregularly Active BA	Regularly Active	Very Active	Non-critical
	Contemplation stage for physical activity practice	Pre-Contemplative	Contemplative	Preparation	Action	Maintenance	Critical
	Feeling of pain	Intolerable	Severe	Moderate	Low	Absent	Critical
	Cardiorespiratory fitness	Very Weak	Weak	Regular	Good	Excellent	Non-critical
	Abdominal strength level	Weak	Regular	Medium	Good	Excellent	Non-critical
	Flexibility level	Weak	Regular	Medium	Good	Excellent	Non-critical
	Manual gripping force	Weak	Regular	Medium	Good	Excellent	Non-critical
**Nursing**	Exposure to environmental risks (physical,	Exposure unknown or higher than limit tolerance, without	Exposure superior to limit tolerance, with protection (EPI/EPC) or	Exposure above NA, with protection (EPI/EPC)	Exposure to irrelevant levels, with or without EPI/EPC	No Exposure	Critical

chemical and biological	individual protection equipment /collective protection equipment (EPI/EPC) protection	above unprotected action level (NA)				
Ergonomic risks—physical aspects	Work place/Work area not assessed by the ergonomics team and not adapted	Work place/Work area not assessed by ergonomics team. Adaptations made by the worker	Work place/Work area assessed by the ergonomics team. Adaptations made by the worker	Work place/Work area assessed and adapted by the ergonomics team	Work place/Work area assessed, adapted by the ergonomics team, in addition to proper posture, the worker refers comfort and safety	Critical
Ergonomic risks—organizational aspects	Organizational factors in intolerable condition. Worker interaction with policies and processes that imply negative interference in health	Organizational factors in tolerable condition. Worker interaction with policies and processes that imply negative interference in health	Organizational factors in tolerable condition. Worker interaction with policies and processes without interference in health	Organizational factors under favourable conditions. Interaction of the worker with policies and processes without interference in health	Organizational factors under favourable conditions. Positive worker interaction with policies and processes contributing to health maintenance	Non-critical
Work environment health conditions	Weak sanitary aspects (Physical structure, hygienic-sanitary conditions, organization of the environment)	Bad sanitary aspects (Physical structure, hygienic-sanitary conditions, organization of the environment)	Good sanitary Aspects (Physical structure, hygienic-sanitary conditions, organization of the environment)	Optimum sanitary Aspects (Physical structure, hygienic-sanitary conditions, organization of the environment)	Same previous condition; Maintenance and Implementation Plan of 5S Organization Tool	Critical

Pests and vectors	Infestation of pests and/or urban vectors with health damage	Infestation of pests and/or urban vectors without harm to health	Presence of pests or urban vectors with partial monitoring	Monitoring the synanthropic fauna of working environments without damage to health	Same previous condition, with monitoring of the annual environmental profile	Non-critical
Air quality	Does not carry out cleaning and maintenance actions of the air conditioning system and/or there is no schedule	Performs cleaning and maintenance of the air conditioning system, but does not have standard operational procedure (POP) and Maintenance, Operation and Control Plan (PMOC), or does not follow the POP or PMOC or schedule, or these are not made available to the executors	Performs cleaning and maintenance actions of the air conditioning system, it has POP, but does not meet all legal requirements and/or PMOC performed by demand or does not make records available.	Performs cleaning and maintenance actions of the air conditioning system, according to the schedule, POP and PMOC, which comply with the legislation and are made available, as well as the records of the execution of the procedures established in the PMOC	Same previous condition, with evidence of periodic trainings of the executors	Non-critical
Drinking water quality	No reports available or reports with illegible results or values that are not in compliance with current legal standards	Reports do not present all the information as required by law	Reports with some illegible information, but do not compromise the results	Reports available, legible and with all parameters and values adequate to legal requirements	Same previous condition, with reports that have been in compliance for more than 6 months	Non-critical
Work accident	Typical work accident, with permanent leave and injury in the last year	Typical work accident, with absence and permanent injury in the last year	Typical work accident, no leave in the last year	No typical work accident history in the last year	No history of work-related accidents in the last 5 years	Non-critical

Work-related absenteeism	Retirement related to work between 16 and 60 days in the last year	Work-related leave less than 15 days in the last year	No history of work-related absence in the past year	No history of work-related absence in the past year	No history of work-related absence in the last 5 years	Non-critical
Family relationships	Conflicting family environment components that negatively affect health, have been identified	Mobilizing components of the family environment, which negatively interfere in health, have been identified	Mobilizing components of the family environment, that do not interfere in health, have been identified	Stable family environment components that positively interfere in health, have been identified	Same previous condition, with a network of mutual support, which positively interferes in the health of the whole family	Non-critical
Social aspects—leisure	Does not use leisure or social environments as a way of maintaining/recovering health. Does not identify the influence of these actions for well-being	Does not use leisure or social environments as a way of maintaining/recovering health, identifies the importance of these actions for well-being	Uses leisure and social environments as a way of maintaining/recovering health and does not identify the importance of these actions for well-being	Uses leisure and social environments as a way of maintaining/recovering health and identifies the importance of these actions for well-being	Same previous condition. Describes positive health outcomes	Non-critical
Self-care level	Pre-contemplation (Does not implement self-care, is not willing to make changes to improve health condition)	Contemplation (Does not implement self-care, recognizes the need to make changes to improve health condition)	Preparation (Decides to implement self-care, ready to make changes to improve health condition, in the near future)	Action (Implements self-care and makes changes even in the initial phase, to improve health condition)	Maintenance (Implements continuously)	Critical

	Tobacco use	Smoker, 25 or more cigarettes/day	Smoker ,15–24 cigarettes/day	Smoker, 1–14 cigarettes/day	Ex-smoker	Ex-smoker for more than 5 years	Critical
	Stress level and symptoms	Above 7 signs of stress	6 signs of stress	5 signs of stress	4 signs of stress	Less than 3 signs of stress	Critical
	Dyslipidaemia	LDL greater than 190	LDL between 160 and 189	LDL between 130 and 159	LDL between 100 and 129	Below 100	Critical
**Medicine**	Diabetes mellitus (DM)	Glycated Hb > 9% or Fasting glycemia > 200 mg/dl, maintaining these results in the last 2 years and/or installed complication	Glycated Hb > 9% or Fasting glycemia > 200 mg/dl	Glycated Hb between 7% and 9% or fasting glycemia between 130 and 200 mg/dl	Glycated Hb < 7% or Fasting Glycemia < 130 mg/dl	Glycated Hb < 7% or Fasting Glycemia < 130 mg/dl, maintaining these results in the last 2 years	Critical
	Systemic Arterial hypertension (SAH)	SAH with associated clinical conditions (cardiovascular disease or chronic kidney disease)	HAS with presence of target organ injury or DM	HAS with 3 or more risk factors	HAS with 1 to 2 risk factors	HAS without risk factor	Critical
	Musculoskeletal pathology	Symptomatic pathology involving rehabilitation	Symptomatic pathology involving reduction of work ability capacity or restriction	Symptomatic pathology without impact on work ability or symptoms without diagnosis	Asymptomatic pathology with treatment (medication or physiotherapy)	Asymptomatic pathology without treatment	Critical
	Psychiatric pathology	Symptomatic pathology involving rehabilitation	Symptomatic pathology involving reduction of work	Symptomatic pathology without impact on work ability	Asymptomatic pathology with	Asymptomatic pathology	Critical

			ability capacity or restriction	or symptoms without diagnosis	treatment (medication or physiotherapy)	without treatment	
	Altered glycemia	Fasting glycemia > 200 mg/dl, maintaining these results in the last 2 years	Fasting glycemia > 200 mg/dl	Fasting glycemia 126–200 mg/dl	Fasting glycemia 100–125 mg/dl	Fasting glycemia < 100 mg/dl	Critical
	Altered blood pressure	Fasting glycemia < 100 Blood Pressure Stage 3: PAS > 180 and / or PAD ≥ 110 mg/dl	Blood Pressure Stage 2: PAS 160–179 and/or PAD 100–109	Blood Pressure Stage 1: PAS 140–159 and/or PAD 90–99	Border Blood Pressure: PAS 121–139 or PAD 81–89	Normal Blood Pressure: PAS ≥ 120 or PAD ≤ 80	Critical
**Nutrition**	Energy balance intake	Intake ≤ or ≥ 1000 Kcal of energy value	Intake ≤ or ≥ 750 Kcal of energy value	Intake ≤ or ≥ 500 Kcal of energy value	Adequate energy intake (VET)	Same previous condition, with healthy meals	Non-critical
	Simple carbohydrate intake	Intake ≥ 16% of VET	Intake ≥ 13% of VET	Intake ≥ 10% of VET	Adequate simple carbohydrate intake (< 10% of VET)	Eventually consumes simple carbohydrate	Critical
	Saturated lipids intake	Intake ≥ 10% of VET	Intake ≥ 8.5% of VET	Intake ≥ 7% of VET	Adequate saturated lipids intake (< 7% of VET)	Eventually consumes saturated lipids	Critical
	Sodium mineral intake	Consumes more than 5 g of sodium daily	Uses more than 3.5 g of sodium daily	Uses more than 2 g of sodium daily	Adequate mineral sodium daily intake (2 g sodium daily)	Does not add salt to the preparations after they are ready, and when consuming processed foods prioritizes those of low sodium content	Critical

	Fibre intake	Intake less than 22 g/day	Intake less than 23.5 g/day	Intake less than 25 g/day	Minimum consumption of 25 g/day	Adequate fibre intake, with consumption of soluble and insoluble fibres	Non-critical
	Alcohol use	Frequent heavy drinker (drinks 1 time or more per week and consumes 5 or more doses per occasion, once a week or more)	Frequent drinker (drinks once a week or more and may or may not consume 5 or more doses at least once a week, but more than once a year)	Less frequent drinker (drinks 1 to 3 times a month and may or not drink 5 doses or more at least once a year)	Non-frequent drinker (drinks less than once a month, but at least once a year and does not drink 5 or more doses at one time)	Abstemious (drinks less than once a year or has never drunk in life)	Critical
	Level of food knowledge	Unsatisfactory level of knowledge	Bad level of knowledge	Regular level of knowledge	Good level of knowledge	Excellent level of knowledge	Non-critical
	Body weight condition	≥40.0 Severe obesity or BMI <16 (Severe thinness)	BMI 30-39.9 Obesity or 16 to <17 (Moderate thinness)	BMI 25-29.9 Overweight or pre-obese or 17 to <18.5 (Slight thinness)	BMI between 18.5–24.9 Normal or eutrophic	Same previous condition with protective factors of healthy eating and regular physical activity for more than 1 year	Non-critical
	Altered triglycerides	Very high (≥500 mg/dl)	High (Between 200 and 499 mg/dl)	Borderline (Between 150 and 199 mg/dl)	<150 mg/dl	Less than 150 mg/dl with control of saturated fat and sugars intake in the diet	Critical
**Dentistry**	Oral hygiene quality	Bad oral hygiene (oral hygiene index - IHOs > 3.0)	Poor oral hygiene (IHOs from 2.1 to 3.0)	Regular oral hygiene (IHOs from 1.1 to 2)	Adequate oral hygiene (IHOs from 0 to 1)	Adequate oral hygiene with bi-annual basic preventive treatment	Non-critical

Periodontal condition	Community periodontal index (IPC) 3 (periodontal bag of 4–5 mm) or 4 (periodontal bag >= 6 mm)	IPC 2 (calculus presence)	IPC 1 (presence of bleeding probing)	IPC 0 (Healthy gum)	Same previous condition with adequate oral hygiene	Critical
Bruxism	Visible wear with dentine exposure with loss greater than 2/3 of the clinical crown, associated with the presence of one more bruxism sign/symptom	Visible wear with dentine exposure with loss greater than 1/3 to 2/3 of the clinical crown, associated with the presence of one more bruxism sign/symptom	Visible wear with exposure of dentin and loss of up to 1/3 of the clinical crown, associated with the presence of one more bruxism sign/symptom	Visible wear restricted to enamel associated or not with the presence of one more bruxism sign/symptom. With use of myorelaxant plaque	Same previous condition with multidisciplinary follow-up	Non-critical
Periodontal disease	Untreated periodontitis, with dental mobility, associated with risk factors (smoking and/or chronic non-communicable disease - DCNT)	Untreated periodontitis, with dental mobility, not associated with risk factors (smoking and/or DCNT)	Untreated periodontitis, without dental mobility, associated or not with risk factors (smoking and/or DCNT)	Treated periodontitis, associated or not with risk factors	Same previous condition associated with the periodic maintenance consultation	Critical
Caries	Presence of caries in dentin, with extensive coronary destruction, and probable involvement of the pulp	Presence of caries in dentin	Presence of active caries, limited to enamel, with or without cavitation	Caries, diagnosed in previous periodic examination, treated	Same previous condition with adequate oral hygiene	Critical

	Oral lesion on soft or hard tissues	Soft tissue lesion with malignant potential, recurrent	Soft tissue lesion with malignant potential	Soft tissue lesion without potential for malignancy	Lesion diagnosed on previous examination, treated	Lesion diagnosed on previous examination, treated, without diagnosis of malignancy	Critical
